# Testing for local adaptation in brown trout using reciprocal transplants

**DOI:** 10.1186/1471-2148-12-247

**Published:** 2012-12-18

**Authors:** Rike B Stelkens, Manuel Pompini, Claus Wedekind

**Affiliations:** 1 Department of Ecology and Evolution, Biophore, University of Lausanne, Lausanne, CH-1015, Switzerland; 2 Institute of Integrative Biology, University of Liverpool, Liverpool, L69 7ZB, UK

**Keywords:** Local adaptation, Reciprocal transplants, *Salmo trutta*

## Abstract

**Background:**

Local adaptation can drive the divergence of populations but identification of the traits under selection remains a major challenge in evolutionary biology. Reciprocal transplant experiments are ideal tests of local adaptation, yet rarely used for higher vertebrates because of the mobility and potential invasiveness of non-native organisms. Here, we reciprocally transplanted 2500 brown trout (*Salmo trutta*) embryos from five populations to investigate local adaptation in early life history traits. Embryos were bred in a full-factorial design and raised in natural riverbeds until emergence. Customized egg capsules were used to simulate the natural redd environment and allowed tracking the fate of every individual until retrieval. We predicted that 1) within sites, native populations would outperform non-natives, and 2) across sites, populations would show higher performance at ‘home’ compared to ‘away’ sites.

**Results:**

There was no evidence for local adaptation but we found large differences in survival and hatching rates between sites, indicative of considerable variation in habitat quality. Survival was generally high across all populations (55% ± 3%), but ranged from 4% to 89% between sites. Average hatching rate was 25% ± 3% across populations ranging from 0% to 62% between sites.

**Conclusion:**

This study provides rare empirical data on variation in early life history traits in a population network of a salmonid, and large-scale breeding and transplantation experiments like ours provide powerful tests for local adaptation. Despite the recently reported genetic and morphological differences between the populations in our study area, local adaptation at the embryo level is small, non-existent, or confined to ecological conditions that our experiment could not capture.

## Background

Natural selection can lead to local adaptation, allowing individuals to have higher relative fitness in their native habitat compared to non-native individuals [1,2]. As a consequence, selection against immigrants can reduce gene flow between populations [3,4], which can lead to divergence without physical isolation [5-7]. But evidence for local adaptation is hard to come by. It usually requires a sound knowledge of the populations’ ecology, the prevailing selection agents, and ideally the underlying genetic basis of phenotypic adaptations. An alternative way, not requiring much preexisting knowledge, is reciprocal transplant experiments [8-11]. Reciprocal transplants can also help to disentangle local adaptation (in the sense of genetically based phenotypic divergence) from phenotypic plasticity (in the sense of differential reactions norms).

Local adaptation in salmonid fishes seems to occur with high frequency [12] and a recent review showed that in 55-70% of all comparisons, native populations outperformed non-natives [8]. Even in populations separated by only small geographic distances, local adaptation has been suggested to contribute to adaptive divergence [13-17]. While these studies suggest the possibility of local adaptation, they could only weakly infer it since no reciprocal transplants were performed. Experiments testing for local adaptation in salmonids in a natural setting are generally rare for practical reasons (dispersal, invasiveness of non-natives, recapture difficulties). Thus, most experiments are carried out in common garden settings in hatcheries or in the laboratory [15,16]. The conclusiveness of these experiments is however limited, since the key ecological factors driving local adaptation may not be replicated in the common garden environment and domestication selection may promote genotypes performing well under laboratory or hatchery conditions but not in the wild. To our knowledge, only a few reciprocal transplant experiments in salmonids have investigated fitness (or some proxy of fitness) and identified traits under selection references in 8, [18-21], and only three studies used the populations’ natural habitat as an egg rearing environment [22-24] even though selection in the wild might be substantial during egg incubation and fry emergence mortality may approach 90%; [25]. Considering the growing concern in recent years about the adaptability of temperature-dependent life history traits such as embryonic developmental rates and timing of hatching e.g. [16], local adaptation of salmonids during the embryonic stage has not been sufficiently understood.

**Figure 1:**
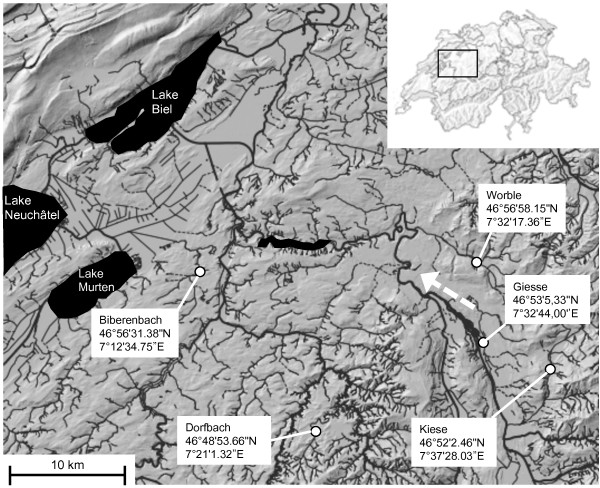
**Schematic map of the egg burial locations within the Rhine drainage system in Switzerland.** GPS coordinates are indicated. Dashed arrow indicates direction of water flow. Box in the upper-right indicates the location within Switzerland. Surface elevation is taken from the *swissALTI3D Reliefschattierung* (Bundesamt für Landestopografie; map.geo.admin.ch).

We used a 5x5 (five populations by five habitats) reciprocal transplant experiment to test for local adaptation in the early life stages of brown trout, raising embryos in their natural streambeds. We used a network of populations inhabiting different tributaries of the Rhine drainage system in Switzerland (Figure 1). To obtain reliable estimates of average population fitness in each of the five environments, we crossed as many parental genotypes as logistically possible in a full-factorial breeding design, i.e. every male was crossed with every female within populations. Artificial insemination helped us to keep the contribution of gametes of each individual balanced. We employed a ‘unique environment’ approach (called ‘unique local adaptation’ approach in [2]), i.e. we had no *a priori* knowledge about the environmental factors potentially acting as divergent selection agents at the five different sites. Embryos were buried in the streambeds in custom-made rearing containers that were used to simulate natural salmonid spawning sites (i.e. redd). At the point where 50% of hatching was predicted to occur (using methods in [26]), we retrieved all embryos from the five locations and recorded survival and the proportion of hatched fry.

We made two predictions with regard to survival: 1) within sites: native populations outperform non-natives; 2) across sites: populations show higher performance at ‘home’ compared to ‘away’ sites. Generally, the first criterion is a better diagnostic for the presence of local adaptation because it is not confounded with differences in intrinsic habitat quality [2]. The first criterion directly tests for the presence of divergent selection within habitats, acting on genetic differences in relative fitness, where non-adapted immigrant genotypes are selected against. The second criterion would only be reliable if all test habitats were of the same quality, which is not usually the case (some habitats may generally cause lower or higher fitness in all populations).

## Results

### Population- specific performance in the laboratory

Fertilization rate in laboratory-raised embryos was high across all populations (Table 1), and no significant differences between populations were found. Thus, any differences in survival in the streams were not caused by variation in fertilization rates between populations. Survival rates until hatching in the laboratory were also high across all populations (Table 1), demonstrating that, under benign conditions, embryos of all populations were capable to successfully finish embryogenesis.

**Table 1:** Location- and population-specific survival rates

		**Lab control**		**Location**					**Population**			
	**N females/males**	**fertilized**	**survived**	**°C**	**day degree**	**survived**	**hatched**	**lost**	**survived**	**hatched**	**lost**	**egg diameter**
Kiese	10/10	97.5%	100%	6.1	517	4.0%	61.9%	2.8%	53.1%	24.0%	2.8%	4.5 ± 0.2
Dorfbach	10/10	94.2%	100%	6.5	522	65.0%	0.0%	1.6%	52.5%	20.3%	2.8%	4.7 ± 0.3
Biberenbach	5/10	92.5%	100%	5.2	494	40.5%	6.0%	0.8%	58.0%	28.4%	2.8%	4.9 ± 0.2
Giesse	8/10	95.4%	97.3%	6.8	472	76.9%	47.4%	4.6%	54.4%	23.7%	1.8%	5.0 ± 0.3
Worble	6/10	94.2%	100%	5.8	463	88.5%	31.5%	1%	58.1%	28.3%	2.6%	5.1 ± 0.2

Number of females and males used to generate embryos, fertilization and survival rates until hatching in the lab-reared control groups, average temperature (°C) and day degrees at each location between time of burial and retrieval, location-specific and population-specific survival rates, rates of hatching among the survivors, and rates of missing eggs at retrieval from streams (‘lost’). The last column shows mean population egg diameter in mm (±SD).

### Population- and site-specific survival in the field

**Figure 2:**
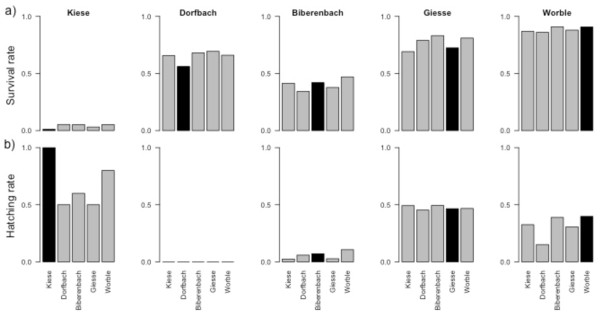
**Survial and hatching rates a) Survival rates at each location (location identity indicated in graph header). b)** Rates of hatched larvae among the survivors at each location. Filled bars indicate the population native at the respective location. Populations are ordered by increasing average egg size. Note that estimates of hatching rates at the Kiese location were based on only few survivors.

Overall survival rates were similar across populations, ranging from 52.5% (Dorfbach) to 58.1% (Worble; Table 1, Figure 2a). Between locations on the other hand, survival rates varied widely, ranging from only 4% (Kiese) to 88.5% (Worble). Populations differed in average egg size (*F*_4,38_ = 5.51, *P* = 0.002; Table 1) with eggs from Kiese females being significantly smaller than eggs from Giesse and Worble females. However, population egg size was not related to mean survival (F _1,5_ = 3.5, p = 0.2).

The full GLMM - using *Population*, *Location*, and *Population × Location* as fixed effects, and *Position* and *Capsule* as random effects - explained significant amounts of variance in survival (Table 2a). No overdispersion was found. Removal of *Population × Location* did not result in a significantly worse or better fit. If local adaptation was present, mean population fitness should be systematically higher for the five native population x habitat combinations than in the remaining cases. The absence of a significant interaction effect suggests that this was not the case. Removal of *Population*, *Location*, *Capsule*, and *Position* (all separately) from the model on the other hand, decreased the goodness of fit significantly. These variables were thus retained in the model. Pairwise Tukey-Kramer comparisons between locations (based on the GLMM without interaction term) showed that survival rates differed significantly between all (−8.20 ≤ *Z* ≥ 3.30, all *P* ≤ 0.008) but two locations (Giesse vs. Dorfbach: *Z* = −2.24, *P* = 0.16). In contrast, none of the pairwise Tukey-Kramer comparisons between populations (based on the GLMM without interaction term) yielded significance, suggesting that, overall, the five populations responded similarly to the environmental conditions at each site.

**Table 2:** Likelihood ratio tests comparing generalized linear mixed models

**Effect tested**	**Fixed effects**	**Random effects**	***AIC***	***X***^***2***^	***d.f.***	***p***
a)						
	**P, L, P×L**	**C, P**	**2149**			
Capsule (C)	P, L, P×L	P	2350	203.1	1	< 0.001
Position (P)	P, L, P×L	C	2169	21.8	1	< 0.001
Population ×	**P, L**	**C, P**	2131	14.2	16	0.58
Location (P×L)						
Population (P)	L	C, P	2135	12.1	4	0.017
Location (L)	P	C, P	2400	277.4	4	< 0.001
b)						
	**P, L, P×L**	**C, P**	**936**			
Capsule (C)	P, L, P×L	P	1061	126.9	1	< 0.001
Position (P)	**P, L, P×L**	**C**	934	0.1	1	0.78
Population ×	**P, L**	**C**	935	9.2	4	0.055
Location (P×L)						
Population (P)	L	C	943	15.8	4	0.003
Location (L)	P	C	942	8.3	1	0.004

(GLMMs) on the effects of population origin, rearing location, position in capsule, and capsule number on a) embryo survival and b) hatching rate among survivors within the locations Giesse and Worble only (because hatching rates or survival were too low in the other populations). Akaikes information criterion (AIC) describes the quality of fit of each model. To evaluate the significance of fixed and random effects, alternative models without the variable of interest were compared to the full model (bold) using likelihood ratio tests. If an alternative model had a significantly better fit (bold), this model was subsequently compared against further reduced models.

In agreement with the above, none of the five separate GLMMs within location (used to test prediction 1) found a native population to outperform non-native populations within location. Similarly, the five GLMMs within population (used to test prediction 2) did not find populations to generally perform better at their ‘home’ than at ‘away’ sites.

We conclude that i) there is no evidence for local adaptation in survival overall, and ii) that locations differed considerably in their quality for egg incubation, causing responses in embryo survival independent of their population origin.

### Population- and site-specific timing of hatching

To compensate for different rates of development caused by temperature differences between streams, we estimated the time when 50% hatching should have occurred for retrieval, taking into account local temperature regimes. However, at two sites hatching had not occurred yet (in Dorfbach and Biberenbach embryos were still alive but had not hatched yet; Table 1 and Figure 2b), and hatching rate at the Kiese was based on only 19 survivors. We therefore excluded these locations and used the two remaining streams (Giesse and Worble) for the analysis of hatching timing. Mean hatching rate across these two sites was 38.8% (Figure 2b). Population egg size was not related to the timing of hatching (F _1,5_ = 1.37, p = 0.33).

The full GLMM explained significant amounts of variance in hatching time (Table 2b). No overdispersion was found. Again, *Population × Location* did not result in a significantly worse or better fit, i.e. there is no evidence for local differentiation in the timing of hatching. Also, the vertical position of the egg within the capsule did not have a significant effect on the timing of hatching of the embryos. Removal of *Population*, *Location*, and *Capsule* on the other hand decreased the goodness of fit significantly and were retained. Tukey-Kramer comparisons between locations (based on the GLMM without the interaction term and *Position*) showed that the timing of hatching differed significantly between the two burial sites (*Z* = −2.94, *P* = 0.003), but only between two of the five populations (Dorfbach vs. Biberenbach: *Z* = −3.69, *P* = 0.002, and Worble vs. Dorfbach: Z = 3.34, P = 0.008; the comparison between Kiese and Dorfbach was marginally significant: *Z* = 2.72, *P* = 0.052).

## Discussion and conclusion

We tested for local adaptation in early life-history traits in brown trout using a 5 × 5 population-by-habitat transplant experiment. Embryos were raised in the natural streambed at ‘home’ and at four ‘away’ sites until 50% hatching was expected to have occurred. We tested for both the ‘local vs. foreign’ criterion (native populations show higher fitness than non-natives) and the ‘home vs. away’ criterion (populations have higher fitness in their own habitat than in other habitats) [2].

We found neither of the two predictions confirmed. The response in embryo survival to environmental conditions was largely independent of population origin, i.e. we found no evidence for local adaptation at the embryonic stage for the trait examined.

There are several possible explanations for the absence of local adaptation. For one, the environments we chose (and the populations sampled) may have not been sufficiently different from each other. Speaking against this, however, our results revealed large differences in survival between sites, ranging from 4% to almost 90%. Since we applied a ‘unique environment’ approach [2], we only assumed that every habitat was distinct and different in its ecological parameters, and no link was established between the prevailing ecological conditions and the phenotypes they select for. Yet, the survival data suggests that sites indeed differed in habitat quality parameters that are crucial to brown trout, such as chemophysical (e.g. temperature, oxygen availability, rate of discharge) and ecological characteristics (e.g. substrate grain size, degree of shading) [27,28]. Alternatively, the microclimates at the burial sites may not have been representative of the habitat conditions typically encountered by the native population and the egg containers we used may not sufficiently simulate the conditions in a natural redd. Furthermore, if mothers exhibited preferences for particular characteristics of their redd, the use of artificial nests could miss aspects of offspring adaptation to the specific incubation conditions locally adapted mothers usually create for them.

In one case (Kiese), conditions seemed unsuitable for egg incubation altogether. Within-stream replication of burial sites would have helped to distinguish between a genuine absence of local adaptation and microclimatic effects. Arguing for the importance of microclimatic conditions during egg incubation, we found strong effects of the individual egg capsules, i.e. the positioning even within one square meter of riverbed made a difference to survival. Also the vertical positioning within the capsule was important, such that eggs closer to the surface had a higher chance of survival than eggs positioned deeper in the riverbed.

There are many environmental threats to an embryo developing in the riverbed. Hatching has been shown to be an inducible defense mechanism in salmonid fishes, such that populations inhabiting risky habitats may hatch earlier or later to escape suboptimal conditions [29,30]. Variation in the timing of hatching can be caused, for example, by desiccation, oxygen shortage, pathogens, or predation. Alternatively, selection may target female spawning time, which can also affect the emergence time of the fry. Whatever the selective mechanism, if populations are genetically adapted to their home site, and if sites differ in selection regimes, differences in the timing of hatching between populations within sites can be expected. Indeed, timing of hatching differed significantly between locations, but all five populations responded similarly to the conditions prevailing at these sites, indicating that, in this population network, the timing of hatching depends largely on the local environmental conditions rather than on population differences.

There are more scenarios that could explain the absence of adaptation in survival and the timing of hatching. For one, maternal investment can potentially confound effects of local adaptation [2,8]. Egg size provides a proxy for the quality of maternal investment (larger eggs contain more yolk) and has been shown to affect growth and survival [31]. Although we could not statistically control for within-population variation in maternal effects since maternity was not tracked until retrieval, we used large numbers of randomly selected dams per population, which should help to obtain reliable estimate of average population fitness. Although populations differed in average egg size, we found no evidence for a direct association between mean egg size and survival or hatching, suggesting that differential maternal investment is not responsible for the absence of local adaptation here.

Both, high levels of gene flow and temporally varying selection can prevent local adaptation [32,33]. However, high gene flow is unlikely in this system. A recent study on brown trout in an area overlapping with our study area found substantial genotypic and phenotypic differences in locomotory and trophic morphology between geographically close populations (2-40 km), arguing for the presence of local adaptation or phenotypically plastic response [17]. Another recent study on brown trout in Switzerland found evidence for adaptation of brown trout to altitude [34]. Generally, brown trout are known to have spatially restricted ranges and genetic differentiation on small geographic scales has been repeatedly reported [35-37]. One possibility is, that population differences in survival due to local adaptation only become evident at later developmental stages in this system.

Although cantonal stocking policies in this area stipulate that streams can only be stocked with offspring from native individuals (further details in [17]), domestication selection can reduce the efficacy of divergent selection and local adaptation. Individuals spending their embryonic and juvenile development in hatcheries are not exposed to natural selection at their site of origin, and the alleles beneficial for survival in the wild may never come to fixation in continuously stocked populations. Hansen *et al.*[38] argued that repeated introduction and admixture of wild and hatchery brown trout in Denmark have led to reduced local adaptation, and hatchery strains of salmonids are often subject to domestication selection [39] with the result that their fitness in the wild is decreased relative to wild fish [40-42]. Some of the adults, used to generate embryos in this experiment, may have been hatchery-raised individuals released into the streams in previous years. Additional file 1: Table S1 shows the stocking effort in the five study streams over the last 30 years. Interestingly, the Worble was stocked with by far the lowest numbers of hatchery-raised fish. At the same time, embryos from the Worble were the only ones performing better at their ‘home’ site than at three other locations. We agree with Fraser *et al.*[8] and Meier *et al.*[39] that more information is needed on how hatchery fish might affect adaptation at local and regional scales.

We conclude that there is a great need to evaluate the prevalence and spatial scales of local adaptation in the wild, and to establish the link between molecular variation and variation in fitness in local adaptation research. More integrative approaches and collaboration between disciplines will help to understand the molecular basis of local adaptation, and how it may vary with developmental stage, selection gradients, geographic scale, and demography.

## Methods

### Choice of test streams and egg burial locations

The five test streams were part of a hatchery’s yearly spawner collection: every autumn, the state hatchery in Reutigen collects adult breeders from different rivers and streams of the Berne canton, and uses them for supportive breeding, i.e. males and females are stripped of their gametes in the hatchery, embryos are produced by artificial insemination, and the fry are released into their streams of origin in the following spring or summer. Geographical distance between the test streams was maximized (within the sample of streams available) to increase the likelihood that ecologically and genetically distinct populations were sampled. A recent study on brown trout in an area overlapping with the area here, showed that populations can differ significantly in both genotype and phenotype on a scale of 2-40 km [17]. Another criterion for stream selection was that the beds of the stream had to be accessible and the substrate suitable for the burial of egg capsules (see below for further details). Egg burial took place in locations where natural spawning sites had been observed in previous years by hatchery staff (pers. comm. Ulrich Gutmann, Fisheries Inspectorate Bern; see GPS coordinates in Figure 1).

### Sampling of populations and fertilization protocol

In November 2010, sexually mature *S. trutta* males and females were collected within a 200 m stretch up - and downstream from the five determined egg burial sites (Figure 1). Animals were collected by electrofishing and transported to the hatchery where they were held until further use. Note that for analysis purposes, individuals collected at the same site are assumed to belong to the same ‘population’ although they may represent a sample rather than a biological population.

On December 1st 2010, fish were anesthetized with clove oil. Before fertilization, ten eggs from each female were photographed in a Petri dish on graph paper. Egg diameter was measured digitally in ImageJ 1.44 to the nearest 0.01 mm. Three measurements per egg were taken from five eggs of each female and averaged to obtain estimates for egg size (Table 1).

**Figure 3:**
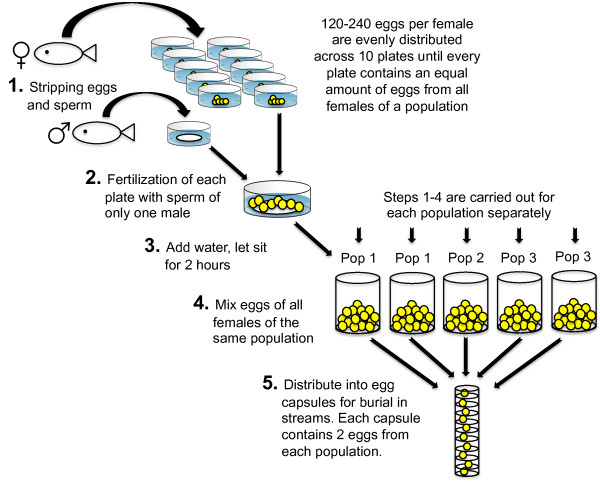
**Flow chart illustrating the full-factorial breeding design.** Sample sizes of males and females used per population can be found in the Methods .

The following fertilization protocol was applied separately for each of the five populations (Figure 3). Ten males and five to ten females (depending on availability, see sample sizes in Figure 1) were stripped off their gametes into separate sterile containers. The unfertilized eggs of each female were carefully distributed in even numbers across ten sterile Petri dishes (Ø 90 mm) with a paintbrush, until each dish contained exactly the same number of eggs from all females. Fertilization in each dish was induced by adding 20 μl of milt of only one of the ten males to avoid potential sperm competition and uneven fertilization rates per male. About 15 ml of sterile, oxygenated water standardized following OECD guidelines; [43] were added to activate the sperm, and dishes were gently agitated to mix gametes. After about five minutes, another 50 ml of water were added and fertilized eggs were left undisturbed for 2 h to promote egg hardening. Then, all fertilized eggs of the same population were mixed thoroughly resulting in a balanced sample of male and female breeder identity. The paternity and maternity of embryos remained unknown hereafter, while population identity was tracked until the end of the experiment. This fertilization scheme allowed for full-factorial breeding within populations (every female was crossed with every male). At the same time, we maximized the number of parental genotypes (a total of 89 breeders was used, yielding 310 full-sib families across populations that were each tested at 5 different locations and under benign conditions in the laboratory, see below). The resulting high genetic variation in offspring genotypes should then provide good estimates of average population fitness in different environments. The number of replicate eggs per sibship ranged from five to ten eggs, depending on how many females were available per population (using more eggs per sibship would have made the design unmanageably large). The alternative, i.e. using fewer breeders with more replicate eggs per sibship, would have given more exact fitness estimates of particular male - female combinations, but such quantitative genetic estimates were not the aim of this experiment.

For burial in the natural riverbeds, a total of 2,500 fertilized eggs (500 eggs per population) were distributed into 250 custom-made egg capsules constructed from stainless steel mesh wire, bolts and washers (Ross-Gillespie *et al. in prep*). Each capsule consisted of ten equally sized, vertically arranged compartments, each for one egg. The mesh wire allowed for good through flow of water in the stream while keeping embryos separate and identifiable. Eggs were distributed systematically, such that each capsule contained two eggs per population, filling each vertical position within the capsule with equal frequency across populations (i.e. yielding 50 replicate capsules with five different distribution schemes). On December 2nd 2010, capsules were transported in chilled standardized water to the five burial sites (Figure 1) and inserted into the gravel using a customized metal tool (similar to a pottiputki tree planting tube). A total of 50 capsules were buried at each site in rows of five capsules, keeping a distance of ca. 10 cm between capsules and rows. Each of the five burial sites received ten replicate capsules of each of the five distribution schemes, making sure that each population was represented in equal numbers in every capsule and every vertical position at each site. In summary, we allocated 100 eggs of each population to 1) their native spawning site, and 2) to the native spawning sites of the four other populations. Buried eggs remained in the stream until their retrieval between February and March 2011.

Digital data loggers were installed at each site (iLog, ESCORT data loggers Inc., USA), recording water temperature hourly until the end of the experiment. On February 6th 2011, data from each logger were downloaded and loggers were placed back in the streams. Eggs were left untouched. The average stream temperature until February 6th was then used to predict the time until 50% hatching, using the method provided in Elliott and Hurley equation 1a; [26], with the aim to retrieve all embryos at approximately the same degree day (to be able to compare survival and hatching rates between sites). After including the remaining temperature data (from February 6th to retrieval), capsules from all sites were retrieved at 493 ± 26 degree days (between 69 and 80 actual days) after fertilization (Table 1). Eggs were visually inspected in the field directly after removal from the riverbed and scored for survival and hatching. Individuals alive at the time of retrieval were considered *survived* (0 = dead, 1 = alive, both unhatched and hatched individuals). Individuals that had fully emerged from their chorion were considered *hatched* (0 = alive and unhatched embryos, 1 = hatched embryos). The specific causes of death could not be assessed in this study, but viral, bacterial and fungal pathogens as well as oxygen deprivation or pollution can be considered potential agents. In case of death, alevins were mostly visible inside the egg, and often in an early eyed stage.

Overall, 64 embryos (2.6% of 2,500 eggs) were missing at the time of retrieval, i.e. the compartment within the egg capsule was found empty. In two cases (Kiese and Giesse), an entire capsule was not found (Table 1). All missing embryos were excluded from further analysis.

Because embryos could not be constantly monitored in the streams and initial variation in fertilization success between populations can potentially distort measures of population-specific survival, fertilization success was monitored in the laboratory under sterile and temperature-controlled conditions as follows. On December 1st 2010, a subset of 240 eggs of each population was distributed singly into the wells of 24-well cell culture plates, which contained 2 ml per well of sterile, standardized, aerated water. Culture plates were transported in a chilled cooler box to a climate chamber at the University of Lausanne (Switzerland) where the embryos were kept undisturbed at constant 6.5°C. Every 3 days eggs were checked under the stereoscope for embryos from three weeks to seven weeks after fertilization had occurred (Dec 21st 2010 - Jan 18th 2011). If no developing embryo was visible by the end of this period, eggs were scored as not fertilized. Survival rates were recorded once hatching (or death) had occurred.

### Statistical analyses

The conclusiveness of reciprocal transplant experiments increases with the dimension of the population-by-habitat matrix (i.e. the number of populations and habitats tested). Generally, more than two populations are needed to differentiate effects of local adaptation from the effects of drift or migration, causing similar population-by-habitat interactions [2], and only replication on the population level allows for identifying fitness differences caused by divergent selection. The larger the transplanting matrix, however, the less likely it is that native populations outperform non-natives in every case, and the prediction must be reformulated such that, across all population-by-habitat combinations, mean population fitness should be higher for natives than for non-natives [2]. We thus tested for differences in performance between populations and burial sites applying a generalized linear mixed model (GLMM) using the Laplace approximation with *Population* (5 levels), *Location* (5 levels), and the interaction *Population × Location* as fixed effects. We also tested for the effects of the position of the embryo in the egg capsule (*Position* ranging from 1 to 10), and capsule number (50 capsules per location), included as random effects. We used a binomial fit for the binary response variables *survival* and *hatching*. To evaluate the significance of fixed and random effects, alternative models without the variable of interest were compared to the full model using likelihood ratio tests. If an alternative model had a significantly better fit, this model was subsequently compared against further reduced models. To test for overdispersion, all variables were treated as fixed effects (converting to a GLM for this purpose), and the residual deviance of the model was divided by the number of degrees of freedom (which should approach 1 in case of no overdispersion [44]). Tukey-Kramer posthoc tests were applied to test for significance of pairwise comparisons between populations and locations using the *multcomp* package [45].

To specifically test our first prediction (native populations outperform non-natives within sites) we ran another five GLMMs on survival, separately per location, using *Population* as fixed effect and *Capsule* and *Position* as random effects. To test our second prediction (populations outperform others at ‘home’ but not at ‘away’ sites) we ran five more GLMMs on survival, separately per population, using *Location* as fixed effect and *Capsule* and *Position* as random effects.

ANOVA was used to test for population differences in egg size and for associations between mean population egg size and survival or the timing of hatching. All statistical analyses were performed in R (version 2.9.2; http://www.r-project.org) using the lme4 package [46].

## Competing interests

The authors declare that they have no competing interests.

## Authors’ contributions

RBS conceived the study, designed and carried out the experiment, analysed the data and drafted the manuscript. MP participated in carrying out the experiment, and analysed the data. CW participated in designing and carrying out the experiment, and guided and reviewed the writing of the manuscript. All authors approved the final version of the manuscript.

## Supplementary Material

Additional file 1: Table S1 Summary of the hatchery program for brown trout in the five study streams. Alevins were raised for some weeks (either in the hatchery or in small streamlets) before released into the stream of origin. Click here for file
