# Supplementary material for: Testing for local adaptation in brown trout using reciprocal transplants
Source: BMC Evol Biol. 2012 Dec 18;12:247. doi: 10.1186/1471-2148-12-247 (PMC3567948; doi:10.1186/1471-2148-12-247)
Supplement: Additional file 1: Table S1 — Summary of the hatchery program for brown trout in the five study streams. Alevins were raised for some weeks (either in the hatchery or in small streamlets) before released into the stream of origin. [file 1471-2148-12-247-S1.pdf]

Supplementary Material

Table S1: Summary of the hatchery program for brown trout in the five study streams. Alevins were raised for some weeks (either in the hatchery or in small streamlets) before released into the stream of origin.

Stream	n years[1]	n females per year [2]	n alevins per year [2,3]	Total n of alevins produced for stocking[3]
Kiese[4]	22	103.3 (6-266)	32.8 (2.8-66.5)	721
Dorfbach	11	136.8 (89-230)	52.2 (25.5-88.6)	574
Biberenbach	24	90.6 (45-181)	34.7 (18.3-100.3)	832
Giesse[4]	18	54.7 (15-99)	38.7 (5.6-76.1)	697
Worble[4]	14	75.7 (17-189)	33.4 (6.6-73.5)	234

[1] number of years that spawners were collected from the respective stream between winter 1981/82 and 2010/11

[2] mean and range

[3] in thousands

[4] data extracted from Stelkens *et al.* (2012)
